# The Maybrick Case: Lawyers v. Doctors

**Published:** 1889-08-17

**Authors:** 


### The Hospital

A Weekly Institutional Journal of

Science, Medicine, Nursing, and Philanthropy.

*For Hospitals, Asylums, Medical Practitioners, Students, Nurses, Families, Parishes, Congregations, and the Charitable Public.*

Vol. VI. No. 151.] [August 17, 1889.

### THE MAYBRICK CASE: LAWYERS v. DOCTORS.

In the course of his summing-up of the evidence in the Maybrick case, Mr. Justice Stephen reminded the jury that "a doctor had been defined as a man who passed his time in putting drugs of which he knew little into a body of which he knew less." One of the penalties a doctor pays for the choice of his profession is that of playing the part of a target for all and sundry to shoot at. He is by no means alone in the performance of that function. Both lawyers and priests are considered fit subjects for the gibbet and the pillory. It is said that when you meet your lawyer you may wish him "good morning," but you must on no account ask him how he is, or you will find six-and-eightpence charged in his next bill for giving you an answer. A lawyer has been defined as a "predatory pleader who plucks pigeons by precedent and pockets the pelf." Whilst "three removes are as bad as a fire," one law-suit may be the ruin of a lifetime. If all the quips and epigrams which dramatists, novelists, and others of the literary craft have written against lawyers could be gathered together, a very clever, very amusing, very uncomplimentary, and very considerable volume would be the result. Similarly, the priest and the parson have always been considered fair game. The popular saying that "if there be a fool in the family he must go into the Church" expresses what used to be a very current opinion in days by no means remote; and the advice put into the clergyman's mouth when he urges his hearers to "do as I say and not as I do," gives point to the common suspicion that the preacher sometimes, at any rate, forgets to put his own doctrines into practice.

The truth is that whenever a man is separated and distinguished from the great mass of his fellow men in any way whatever, from that moment he becomes an object of criticism. The criticism will be good-natured or ill-natured according to the peculiar experience and mental proclivities of the critic. So much of it as happens to be specially witty, or specially appropriate, or specially ill-natured will survive; sometimes by its truth, but more frequently by its wit or its spitefulness; and there will thus be gradually accumulated a sort of "proverbial philosophy" of every profession, into which whosoever is so disposed may dip at any convenient time, and from which he may extract such a proverb or definition as he thinks will suit his purpose at the moment. Mr. Justice Stephen put his hand into the bag of medical proverbial philosophy last week, and drew forth exactly such a keen and caustic epigram as suited his own keen and caustic fancy. We do not complain of Sir James Stephens' choice of a definition. If the medical history of the past forty years has not been such as to render the repetition of so stale a joke powerless for harm, then good work well done may never again hope to wipe out a stigma so long as the world stands.

Into the merits of the Maybrick case we do not propose to enter; but the facts, as they affect the medical men who gave evidence, and indirectly the whole profession, are well worthy of consideration. Whoever wishes to do justice to the doctors engaged in the case throughout will not be unwilling to give due weight to one or two important considerations. It is said there has been a great conflict of medical testimony. Nothing of the kind. There has been a conflict of medical opinion, but no disagreement whatever as to the testimony concerning the actual circumstances. For what were the main facts connected with Mr. Maybrick's death? They were two: the one being that the cause was gastro-enteritis, or inflammation of the stomach and bowel; the other that arsenic was unquestionably found in certain organs of the body after death, and, as Dr. Stevenson, the analyst, said at the time of death, in larger quantities than had sometimes been discovered in undoubted cases of arsenical poisoning. Now, will any one deny that these were the essential facts of the case? If there had been no gastro-enteritis, and no arsenic found in the system, would it have been possible for any counsel whatever to have claimed a verdict of arsenical poisoning? But if these facts constituted the absolute core and essence of the case, to whom was the merit due of bringing them to light? And not only to light, but into such clear and convincing light that no question was ever raised as to their validity? They were concurred in by the counsel and witnesses for the defence as freely as by the counsel and witnesses for the prosecution; by the judge and by the jury, as well as by the public and the press. But the discovery and proof of these indispensable facts were the work of the doctors, and of the doctors alone. Without doctors it would have been absolutely impossible for the judge, the jury, or the public to have discovered either the gastro-enteritis or the presence of arsenic in the organs. That is to say, without medical science a hundred Mr. Maybricks might be poisoned every day, and no one would be able to say from an examination of the body what the cause of death might have been. When, therefore, it is asked, as it was by the *Pall Mall Gazette*, "Is medical science a failure?" and when it is gravely assumed that in the Maybrick case, at least, it has been so, there is shown as complete a misapprehension of the main points at issue as can well be imagined.

If it be said that the doctors differed in opinion as to the cause of the gastro-enteritis, although they agreed as to the fact, the reply is obvious. Those doctors who attended Mr. Maybrick during life, who made the post-mortem examination, who actually saw the stomach and bowels, and who were able, therefore, to add to their knowledge of the living subject the personal inspection of the organs after death, did not disagree, but were all entirely at one. The other doctors who ventured to differ from them did so on hearsay and report, and not on personal knowledge. They did not, therefore, in any sense fulfil the conditions of scientific requirement as specialists and experts. They did not, in short, deal with facts and first-hand knowledge, but with opinions and knowledge at second-hand. We venture to express the conviction that under the circumstances the medical witnesses for the defence were by no means justified in the strong expression of opinion they indulged in, and that they would have better observed the strict canons of scientific requirement, as well as consulted their own reputations, if they had refused to offer any opinion at all, except on first-hand knowledge. Under the actual circumstances present, the medical opinions brought forward by the defence were not in strict justice entitled to rank as evidence at all. So far as the testimony of the doctors—which was actually valid as real and first-hand evidence—was concerned, there was a practical unanimity of a very striking character.

It may be asked, finally, why was not this view of the case brought out by the judge in his summing-up? The answer is, because of the serious constitutional blunder in our legal system which permits a lawyer, unassisted by medical assessors, to try cases involving facts and circumstances which nobody but a competent medical man can thoroughly understand and correctly value. In almost every case tried by judge and jury where medical questions are involved, there is a conflict of evidence which is scandalous to the medical profession and often fatal to the interests of justice. Why is this? It is because judges, counsel, and juries are all alike equally and completely ignorant of medical questions. If, in addition to the ordinary judge, an eminent medical practitioner were to hear and adjudicate upon medical testimony in every case, such scandals would cease at once; for medical experts would not then come forward to offer mere opinions instead of testimony to facts personally witnessed. In all cases where medical and other questions involving special knowledge are tried, justice is and will continue to be a mere lottery until experts are placed as assessors on the Bench instead of being merely called as opposing witnesses. To set a mere lawyer to try a medical case is just as absurd as to set a mathematician to solve a problem in chemistry, or a captain of dragoons to navigate a man-of-war. Justice may possibly now and again be done in such cases, but it can only be as the result of chance and accident. If Parliament had any adequate capacity for its duty, or medical men as a body more self-respect, the state of things which now obtains would not be allowed to continue for a single year.

